# Supplementary material for: Modulating the CXCR2 Signaling Axis Using Engineered Chemokine Fusion Proteins to Disrupt Myeloid Cell Infiltration in Pancreatic Cancer
Source: Biomolecules. 2025 Apr 30;15(5):645. doi: 10.3390/biom15050645 (PMC12108577; doi:10.3390/biom15050645)
Supplement: Supplementary file 1 [file biomolecules-15-00645-s001.zip › biomolecules-3532169-supplementary.pdf]

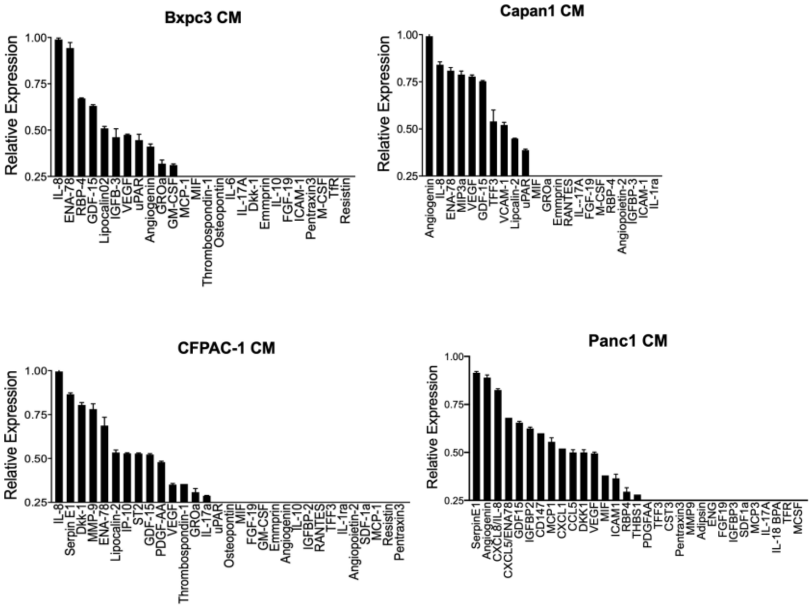

Figure S1.
Cytokine arrays were used to identify relative concentrations of 105 secreted factors from four PDAC cell lines (BxPC-3, Capan-1, CFPAC-1, and PANC-1).

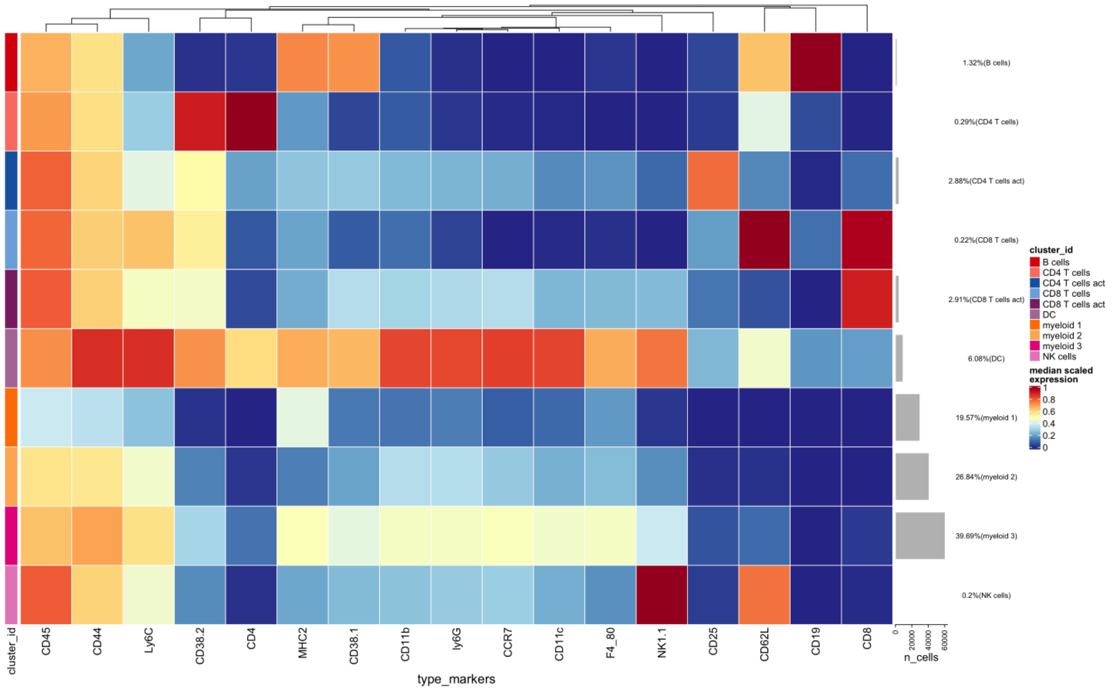

Figure S2.
CyTOF analysis of immune cell subsets were analyzed. Relative expression of the indicated factors and the characterization of immune cell type based on immunophenotype are shown.

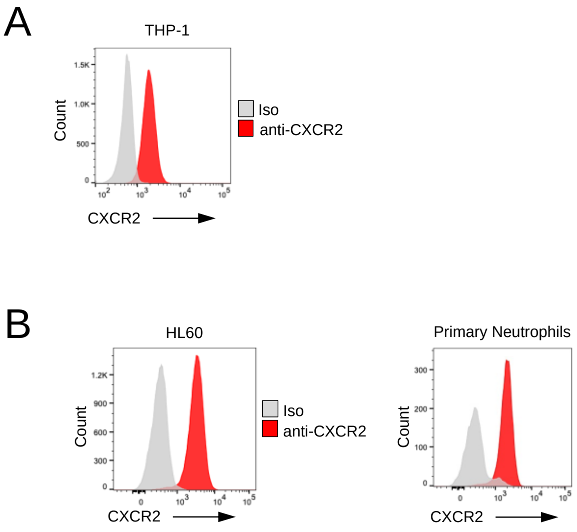

Figure S3.

(A) THP-1 cells were analyzed by flow cytometry using a PE conjugated anti-CXCR2 antibody.

(B) Primary human neutrophils and HL60 cells were analyzed by flow cytometry using a PE conjugated anti-CXCR2 antibody.

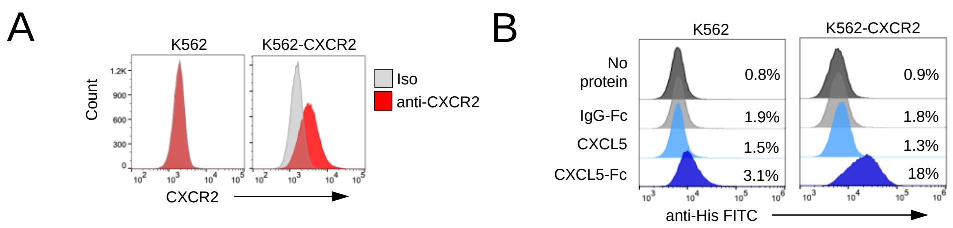

Figure S4.

(A) Parental K562 cells and K562 cells expressing CXCR2 were analyzed by flow cytometry using a PE conjugated anti-CXCR2 antibody.

(B) Parental K562 cells and K562 cells expressing CXCR2 were incubation with Fc, CXCL1, CXCL1-Fc, or PBS as a control and binding was analyzed by flow cytometry using a FITC conjugated anti-His tag antibody.

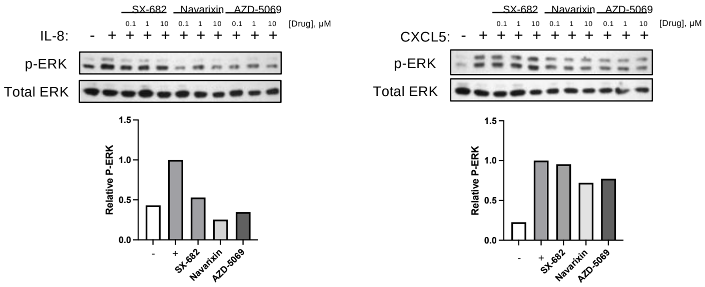

Figure S5.

THP-1 cells were treated with 200nM IL-8 or CXCL5 for 5 minutes in the presence of the indicated concentrations of CXCR2 inhibitors SX-682, Navarixin, or AZD5069. Cells lysates were analyzed by western blotting for p-ERK and total ERK. Original images of Figure 6D can be found in supplementary materials.

A

	Fc	CXCL1-Fc	CXCL1[1]-Fc[2]	CXCL1[1]-Fc[1]
T_m(°C) 95% CI	68.37 ± 0.136	66.47 ± 0.192	67.13 ± 1.347	64.43 ± 0.136

B

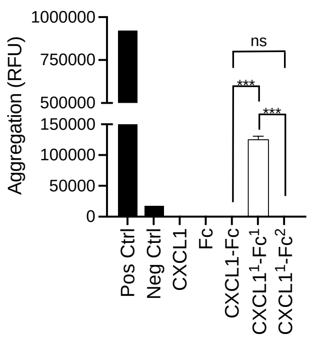

Figure S6.
(A) Fc, CXCL1-Fc, CXCL1[1]-Fc[2], and CXCL1[1]-Fc[1] were analyzed by a thermodynamic stability assay. Data is displayed as melting temperature (T_m). Statistical significance was determined using Ordinary one-way ANOVA and CXCL1-Fc, CXCL1[1]-Fc[2], and CXCL1[1]-Fc[1] were compared by Šídák's multiple comparisons test (* $p < 0.05$, N = 3).
(B) Fc, CXCL1, CXCL1-Fc, CXCL1[1]-Fc[2], and CXCL1[1]-Fc[1] were analyzed by an aggregation assay. Statistical significance was determined using Ordinary one-way ANOVA and CXCL1-Fc, CXCL1[1]-Fc[2], and CXCL1[1]-Fc[1] were compared by Šídák's multiple comparisons test (*** $p < 0.0005$, N = 3).

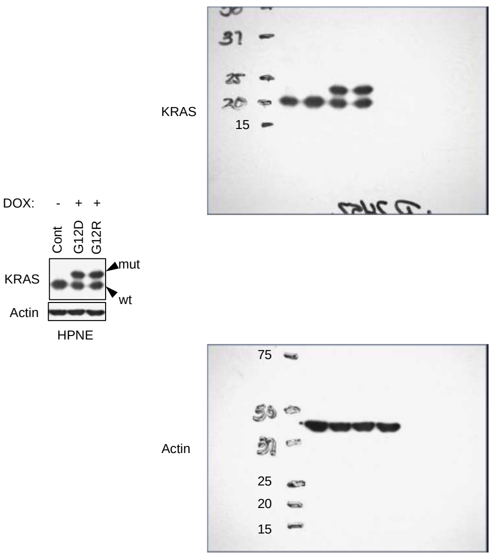

Figure S7.

Original Western blot images of Figure 2A

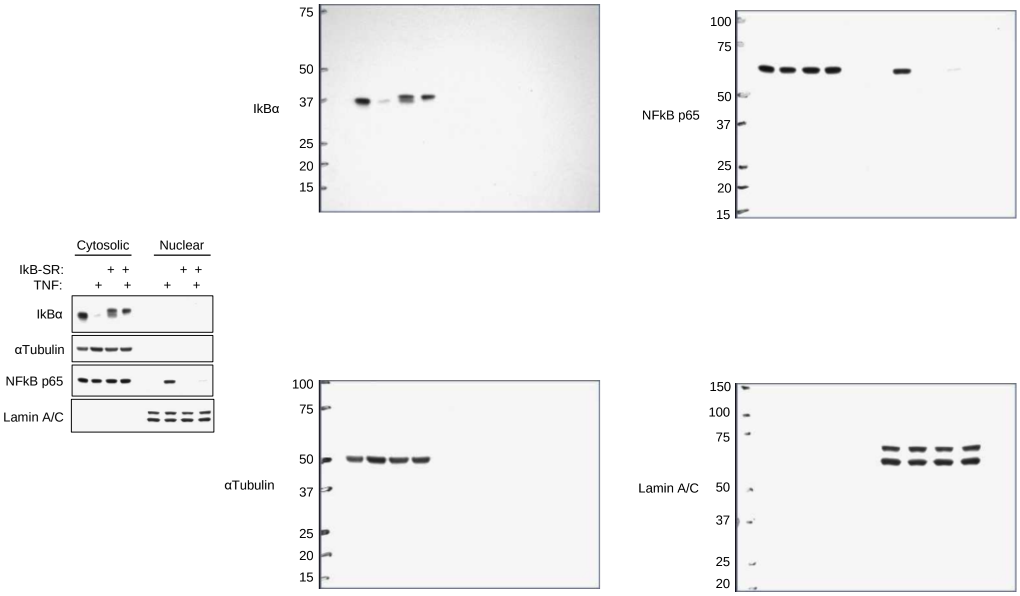

Figure S8.

Original Western blot images of Figure 2D

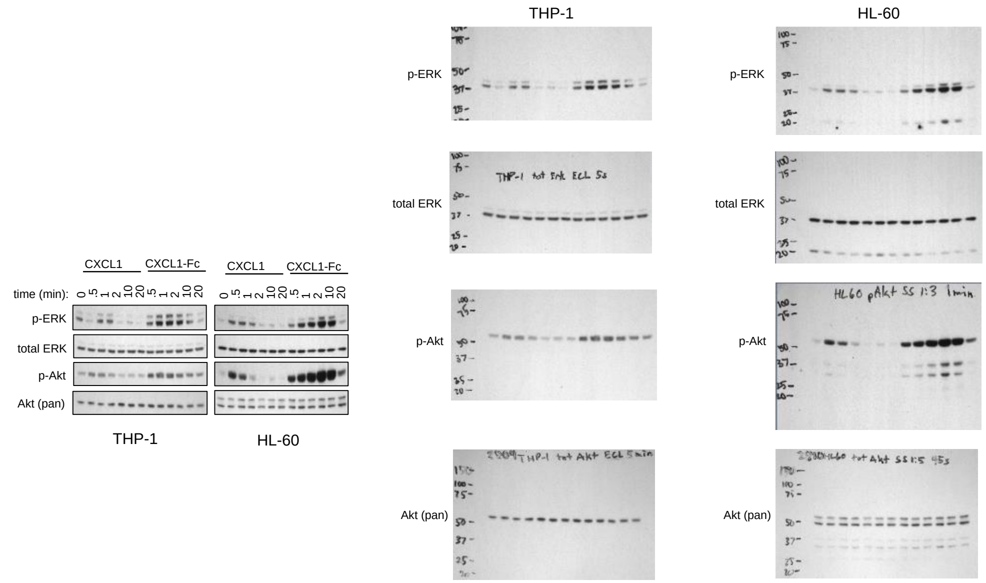

Figure S9.

Original Western blot images of Figure 4E

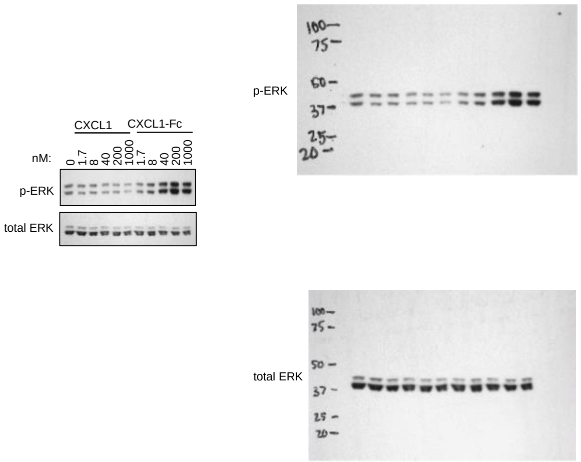

Figure S10.

Original Western blot images of Figure 4F

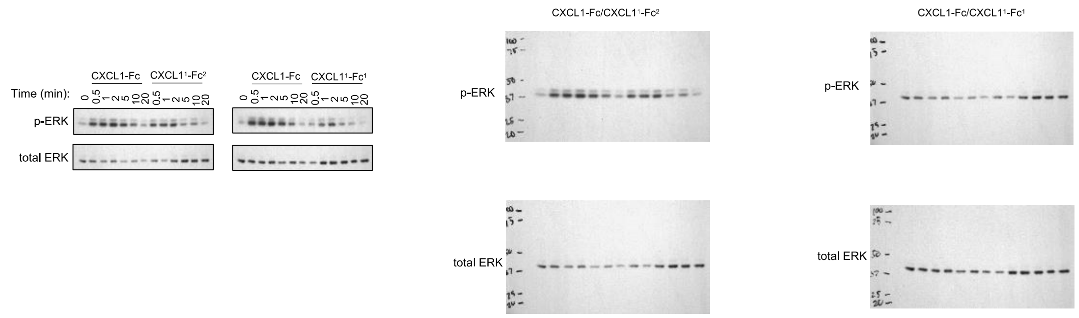

Figure S11.

Original Western blot images of Figure 6D

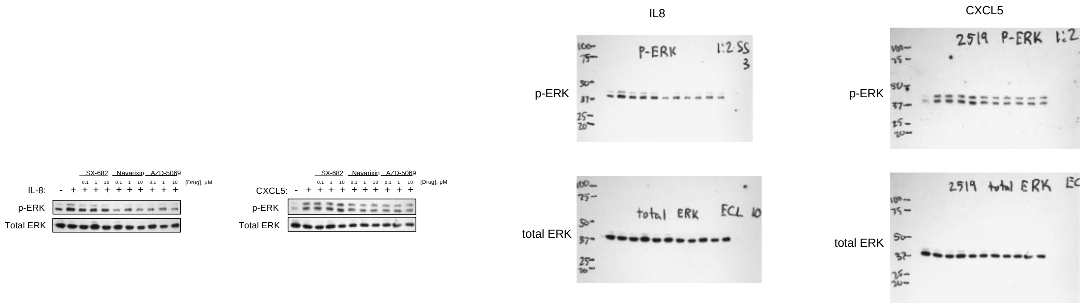

Figure S12.

Original Western blot images of Figure S5
